# Population level changes in schistosome‐specific antibody levels following chemotherapy

**DOI:** 10.1111/pim.12604

**Published:** 2018-12-19

**Authors:** Mizuho Fukushige, Francisca Mutapi, Mark E.J. Woolhouse

**Affiliations:** ^1^ Present address: Faculty of Medicine University of Tsukuba Tsukuba Japan; ^2^ Centre for Immunity Infection & Evolution College of Medicine and Veterinary Medicine University of Edinburgh Edinburgh UK; ^3^ Institute of Immunology and Infection Research Centre for Immunity Infection & Evolution School of Biological Sciences NIHR Global Health Research Unit Tackling Infections to Benefit Africa (TIBA) University of Edinburgh Edinburgh UK; ^4^ Centre for Immunity Infection & Evolution, and Usher Institute of Population Health Sciences & Informatics College of Medicine and Veterinary Medicine University of Edinburgh Edinburgh UK

**Keywords:** antibody levels, human, meta‐analysis, population, praziquantel, schistosomiasis

## Abstract

**Aims:**

Previous studies have reported that chemotherapy of schistosomiasis by praziquantel in humans boosts protective antibody responses against *S mansoni* and *S haematobium*. A number of studies have reported schistosome‐specific antibody levels before and after chemotherapy. Using these reports, a meta‐analysis was conducted to identify predictors of population level change in schistosome‐specific antibody levels after chemotherapy.

**Methods and results:**

Following a systematic review, 92 observations from 26 articles published between 1988 and 2013 were included in this study. Observations were grouped by antigen type and antibody isotypes for the classification and regression tree (CART) analysis. The study showed that the change in antibody levels was variable: (a) between different human populations and (b) according to the parasite antigen and antibody isotypes. Thus, while anti‐worm responses predominantly increased after chemotherapy, anti‐egg responses decreased or did not show a significant trend. The change in antibody levels depended on a combination of age and infection intensity for anti‐egg IgA, IgM, IgG1, IgG2 and anti‐worm IgM and IgG.

**Conclusion:**

The study results are consistent with praziquantel treatment boosting anti‐worm antibody responses. However, there is considerable heterogeneity in post‐treatment changes in specific antibody levels that is related to host age and pre‐treatment infection intensity.

## 1 INTRODUCTION

Schistosomiasis is a water‐borne parasitic disease of great public health importance mainly in sub‐Saharan African countries.^1^ Currently, there is a major effort to have schistosome control programmes in all schistosome endemic countries in Africa.^2^, ^3^, ^4^ There is a need to understand all of the potential effects and implications of schistosome antihelminthic treatment in affected populations. One such potential impact of treatment is on the host immune response against schistosome parasites.^5^, ^6^

It is well documented that naturally acquired immunity against schistosome infections reduces both prevalence and infection intensity in the older age groups in endemic areas.^7^, ^8^ However, this protective immunity takes years of chronic infection to develop naturally. There are two reasons why anti‐schistosome immunity takes long to develop, first; the parasite is capable of evading host immunity and second; the host requires a threshold of antigens to mount a protective immune response. Schistosome worms have an average life span of several years.^9^, ^10^, ^11^ The antigens from adult worms only become accessible to the host immune system following praziquantel treatment.^5^, ^12^, ^13^, ^14^

Praziquantel (PZQ) treatment has been reported to enhance host protective immunity by exposing the parasite's hidden antigens in large amounts,^5^, ^6^, ^15^ and by removing the immunomodulatory effects of adult worms.^16^, ^17^ PZQ treatment allows hosts’ immune systems to recognize schistosome parasite adult worm antigens that is required to develop protective immunity.^15^ In 2001, a review of seven field studies reported high levels of heterogeneity in the type and magnitude of change in antibody levels after chemotherapy between different human populations.^18^ To date, many potential factors have been suggested to explain this variation, such as pre‐treatment infection intensity,^19^ level of schistosome endemicity,^19^ age,^20^, ^21^ sex^21^ and co‐infection with human immunodeficiency virus (HIV).^22^ This is important for understanding the consequences of the epidemiological transition occurring in populations subject to national schistosome control programs through mass drug administration both for the host and the parasite.^23^ However, there has not been a systematic review of published studies investigating the potential determinants of the heterogeneity in post‐treatment immune responses. Therefore, the objectives of this study are (a) to investigate the pattern of antibody levels change across different human populations and (b) to identify host and parasite factors that affect the antibody levels change after PZQ treatment in the population levels.

## 2 METHODS

### 2.1 Systematic review

An electronic literature search was conducted using six databases. Web of Science Core Collection, BIOSIS Citation Index and MEDLINE all of which were searched through Web of Science (www.webofknowledge.com). In addition, EMBASE, Global Health and Ovid Medicine were searched through Ovid (ovidsp.tx.ovid.com). The search terms were as follows: “schistosom*” AND (“antibod*” OR “IgA” OR “IgE” OR “IgM” OR “IgG*”) AND [“albendazole” OR “metrifonate” OR “artesunate” OR “antihelmint*” OR “chemotherap*” OR “praziquantel” OR “oxamniquine” OR (“drug” AND “treatment”)]. This electronic literature search was completed in January 2014. After removing duplicates, a total of 1366 unique articles were identified for consideration in the present study. Titles and abstracts of articles were screened to exclude those that were clearly not relevant. Full texts of potentially relevant articles were then reviewed for further selection. Full texts of the relevant articles were sourced through the Web of Science, Ovid, Google Scholar (scholar.google.com), the University of Edinburgh library and the Inter Library Loan of the University of Edinburgh. Non‐English articles were included in this study, and several Chinese articles were identified and translated into English by a native Chinese speaker for the systematic literature review.

### 2.2 Inclusion criteria

An article was included in this study if it met all of the following criteria: (a) human study (either sex), (b) infection with *S mansoni* and/or *S haematobium* diagnosed by parasitological egg count, (c) participants treated with PZQ, (d) number of participants reported, (e) schistosome‐specific antibody levels reported before and after PZQ treatment, (f) follow‐up study conducted within 14‐180 days after PZQ treatment, (g) schistosome worm antigen (WWA: whole worm homogenate and/or soluble worm antigen), and/or soluble egg antigen (SEA) used to measure antibody isotype levels, (h) participants potentially exposed to schistosome infection for their life‐time and/or longer than 1 year before the study, (i) participants’ ages could be categorized as child (0‐10 years old), adolescent (11‐21 years old) or adult (≥21 years old).

### 2.3 Exclusion criteria

Articles were excluded based on the following exclusion criteria: (a) participants had a previous history of antihelminthic treatment prior to the study participation, (b) participants were treated with any antihelminthic drug other than PZQ (eg, oxamniquine), (c) participants were specifically selected because of co‐infection with HIV and/or soil transmitted helminths and/or plasmodium, (d) purified and/or recombinant schistosome antigens were used to measure antibody isotype levels, (e) participants were temporary visitors to endemic areas (ie, travellers), (f) participants were originally from endemic areas but had moved to non‐endemic areas prior to the study (eg, immigrants), (g) participants were diagnosed with acute schistosomiasis, (h) clinical case reports from a single patient.

In schistosomiasis endemic areas, co‐infection with soil transmitted helminths is frequently reported.^24^ These studies were kept in the analysis only if they did not specifically select co‐infected participants. Schistosome‐specific antibody isotype levels before (baseline) and after (follow‐up) treatment with PZQ were extracted from the selected articles. For those articles that reported results only in graphical format, the software DataThief III (2006) was used to extract the raw data, whenever the graph format allowed it.

**Figure 1:**
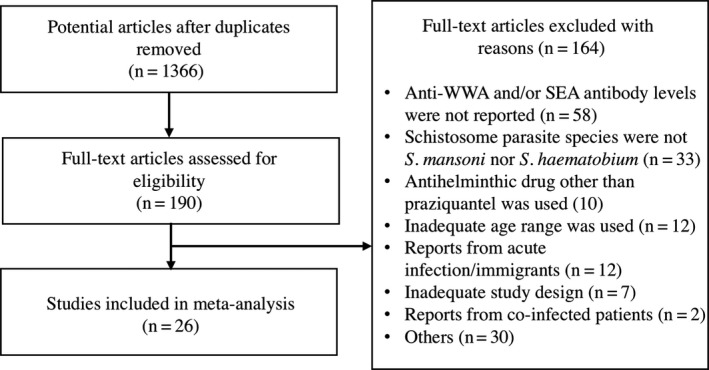
A systematic review flow diagram. Diagram of the number of articles identified and examined at each stage of the review

In addition to antibody levels, the following information was also extracted from each article: the year of publication, article title, names of authors, study area (country, region and village), schistosome parasite species, co‐infection status, co‐infecting pathogen species, number of participants, age or age range, sex, height, weight, days between the treatment and follow‐up, pre‐ and post‐ treatment infection intensity and prevalence, PZQ dose and cure rate. Several articles reported results from multiple different groups of participants in the same study area, such as from different age groups. In such cases, the result from each group was recorded as a separate observation. For the purpose of classification and regression tree (CART) analysis, they were treated as independent observations. For articles that reported results from multiple follow‐up time points, the first follow‐up after 14 days was selected and included in this study. A total of 92 observations from 26 articles (published 1988‐2013) met all the inclusion criteria and were considered for the final meta‐analysis (Figure 1, Tables 1, Appendix S1 and Table S1).

**Table 1:** Summary of 26 articles selected after systematic review

References	Parasite species	Antigen type	Antibody isotype	Follow‐up (days)	No. of participants for each observation and antibody isotype (participants)^a^
Abebe et al. (2001)^21^	S.m	SEA	IgA, IgE, IgG1, IGG2, IgG3, IgG4, IgG, IgM	35	66
Ali et al. (1994)	S.m, S.h	SEA/WWA	IgA	90	21, 51
Feldmeier et al. (1988)^b^	S.m, S.h	WWA	IgE, IgG	150	19
Fouda et al. (2007)^b^	S.m	WWA	IgE	180	19
Grogan et al. (1996) b	S.h	SEA/WWA	IgE, IgG4	35	55
Hamadto et al. (1990)^27^	S.m	SEA/WWA	IgA, IgE, IgG, IgM	49	36, 40
Hussein et al. (1996)^b^	S.m	SEA/WWA	IgG, IgM	60	26, 38
Ismail et al. (1992)^b^	S.m, S.h	SEA/WWA	IgG, IgM	90	20, 15, 14, 11 (SEA)
					10, 8, 7 (WWA)
Joseph et al. (2004)^22^	S.m	SEA/WWA	IgE, IgG1, IgG2, IgG3, IgG4	35	10
Mutapi et al. (1998) (a)^28^	S.m, S.h	SEA	IgA, IgG1	84	132
Mutapi et al. (1998b)^b^	S.h	SEA	IgA, IgE, IgG1, IgG2, IgG3, IgG4	126	37
Nagaty et al. (1996)^41^	S.m, S.h	SEA/WWA	IgA, IgE, IgG, IgM	180	21, 51
Nassr et al. (2002)^b^	S.m	WWA	IgG1, IgG4	90	8
Naus et al. 1998^44^	S.h	SEA/WWA	IgE, IgG1, IgG2, IgG3, IgG4,IgM	30	97
Reilly et al. (2008)^b^	S.h	WWA	IgG1, IgG3	42	89
Satti et al. (2004)^b^	S.m	WWA	IgE, IgG4	21	28
Satti et al. (1996) (a)^36^	S.m	SEA/WWA	IgE, IgG1, IgG2, IgG3, IgG4, IgM	90	17
Satti et al. (1996b)^b^	S.m	WWA	IgA	90	25
Snyman et al. (1997)^b^	S.h	WWA	IgE, IgG	21	14
Snyman et al. (1998)^b^	S.h	WWA	IgE, IgG	90	10, 9
Tweyongyere et al. (2009)^b^	S.m	SEA/WWA	IgE, IgG1, IgG2, IgG3, IgG4	42	89, 124
van Lieshout et al. (1999)^26^	S.m	WWA	IgE, IgG1, IgG3, IgG4, IgG, IgM	63	30
Vereecken et al. (2007)^40^	S.m	SEA/WWA	IgA, IgE, IgG1, IgG2, IgG3, IgG4, IgM	42	21, 24, 61, 143
Walter et al. (2006)^10^	S.m	SEA/WWA	IgA, IgE, IgG1, IgG2, IgG3, IgG4, IgM	35	24, 28 (SEA)
					22, 23, 27, 28, 31 (WWA)
Wilson et al. (2013)^b^	S.m, S.h	WWA	IgE	63	41
Zinyowera et al. (2006)^b^	S.h	SEA/WWA	IgA, IgE, IgG	42	28

S.h, *S haematobium*; S.m, *S mansoni*; SEA, schistosome soluble egg antigen; WWA, whole worm antigen.

a The numbers represent the number of participants for each observation of each antigen time (SEA/WWA) and antibody isotypes (IgA, IgE, IgG1, IgG2, IgG3, IgG4, IgM).

b These references are given in supporting information Appendix S1.

Potential predictors were selected according to their biological importance, as suggested by earlier studies^19^, ^21^ and if they were reported by the majority of articles included in this study. The following predictors were considered: age groups (0‐10 years old, 11‐21 years old, ≥21 years old), pre‐treatment infection intensity [low (*S mansoni*: 1‐99 eggs/1 g faeces, *S haematobium*: 1‐49 eggs/10 ml urine) or high (*S mansoni*: ≥100 eggs/1 g faeces, *S haematobium*: ≥50 eggs/10 mL urine)], schistosome species (*S mansoni*,* S haematobium*, or co‐infection of *S mansoni* and *S haematobium*), disease prevalence (low/moderate: <50% or high: ≥50%), and days between chemotherapy and follow‐up. The “low” infection intensity in the current study is equivalent to “light” in the WHO's infection intensity categories both for *S mansoni* and *S haematobium*. However, “high” infection intensity is WHO's “heavy” for *S haematobium* but “moderate” or “heavy” for *S mansoni*. This was due to the small sample size of *S mansoni* studies: there were only eight observations from four articles that reported >400 eggs/1 g faeces that is “heavy” infection by WHO guideline.

### 2.4 Software

Articles identified by the systematic review were recorded using Thomson Reuters EndNote, and the extracted data were entered in a spreadsheet using Microsoft Excel 2010. B. Tummers, DataThief III. 2006 (http://datathief.org/) was used to extract data from published graphs. IBM SPSS Statistics Version 21.0 was used for statistical analysis. GraphPad Software GraphPad Prism version 6.03 was used to create graphs.

### 2.5 Statistical analysis

The majority of studies included in this study used the enzyme‐linked immunosorbent assay (ELISA) method to quantify antibody isotype levels and reported optical density (OD). However, OD values cannot be directly compared between studies conducted by different research groups.^25^ Therefore, the outcome variable was categorized according to the direction of change in antibody levels from pre‐treatment baseline to levels at follow‐up. That is, pre‐treatment and post‐treatment antibody isotype levels were compared within the same population and the outcome was categorized as “increase” if the post‐treatment level was higher than the pre‐treatment level, and “decrease” if it was lower. There were seven observations that reported the exact same value of antibody isotype levels both pre‐ and post‐ treatment.^10^, ^26^, ^27^ The number of those observations were too small to form their own category “no change”; therefore, they were categorized into “decrease” group in this study for analyses purposes. All observations were grouped according to the type of schistosome parasite antigens (WWA or SEA) that were used to measure antibodies and analysed separately. Prior to the data stratification, we conducted preliminary analyses. The analyses results supported the partitioning by antigen type but not parasite species; therefore, WWA and SEA were analysed separately but not parasite species.

There were 29 observations from four articles that failed to report pre‐treatment infection intensity of study participants. In these cases, pre‐treatment infection intensity was obtained from scientific publications describing the larger populations that contained the study populations (articles listed in Table S2). Similarly, there were three observations from two articles that did not report the schistosome infection prevalence in the study area. In these cases, prevalence was obtained from scientific publications or governmental reports from the same area or larger area that contained the study populations (Table S2).

In schistosomiasis endemic areas, infection intensity peaks in the young age group, giving a convex curve typically observed in schistosomiasis.^28^ Furthermore, our preliminary analyses using a mixed effects logistic regression model suggested that there is an association between age and infection intensity (data not shown). To take this nonlinear association into account, a combination predictor for age and infection intensity was generated, with format age/infection intensity as shown in Table 2. All of the observations were categorized according to age/infection intensity categories. All predictors used for CART analysis are listed in Table 2.

**Table 2:** List of potential predictors investigated and their measurement units/codes

Potential predictors (units)	Codes		
Prevalence (%)	Low/Moderate	High	
	<50	≥50	
Schistosome species	S.m	S.h	Co‐infection of S.m and S.h
Days after chemotherapy (days)	Continuous (14‐180 days)		
Age^a^/infection intensity^b^	Child/low	Adolescent/low	Adult/low
	Child/high	Adolescent/high	Adult/high

S.m, *S mansoni*; S.h, *S haematobium*.

Age category: child (0‐10 y), adolescent (11‐20 y), adult (≥21).

Infection intensity: *S mansoni*: Light 1‐99 eggs/1 g faeces, Heavy ≥100 eggs/1 g faeces, *S haematobium*: Light 1‐49 eggs/10 mL urine, Heavy ≥50 eggs/10 mL urine.

### 2.6 Classification and Regression Tree models

Classification and Regression Tree (CART) models were used to identify influential predictors of the direction of change of schistosome specific antibodies after PZQ treatment.^29^, ^30^ Briefly, CART models are non‐parametric and nonlinear analyses which allow us to investigate the pattern of data without making data distribution assumptions of both outcome and predictors.^31^ CART models do not make assumption about the type of association between dependent variable and influential predictors.^32^ In addition, CART models do not have mandatory minimal sample size that allows us to apply this model to study with a small sample sizes.^32^ Confidence intervals or standard errors are the most common weighting methods for meta‐regression^31^ and for meta‐CART.^33^ However, in this analysis the measures of antibody levels were ELISA OD values, which cannot be compared directly when coming from different research groups.^25^ Therefore, in this analysis, the sample size (the number of participants) was used for weighting. The number of participants across studies varied from 7 to 148. The potential predictors used for the analysis were as follows: schistosome species (*S mansoni*,* S haematobium*, or co‐infection), days between treatment and follow‐up, and disease prevalence (low/moderate or high), and age/infection intensity (child/low, adolescent/low, adult/low, child/high, adolescent/high, adult/high) (Table 2).

The CART analysis was conducted to build a tree using the standard three steps: (a) growing a maximum‐sized tree with largest number of subgroups, (b) pruning the tree to generate subtrees and (c) identifying the optimal sized tree with the minimal risk estimate following the methodology of Breiman.^30^ Initially, the maximum‐sized complex trees were grown with data from all study variables for each antibody isotype. All potential predictors were compared using the Gini index to identify the optimum split of the dependent variable (increase or decrease in antibody level). Based on the Gini index, the strongest predictor variable and its splitting value, that is sub‐groupings for categorical variables and cut‐off values for continuous variables, were used to split the original data (ie, root node) into two subgroups (ie, daughter nodes). The subgroups were then divided repeatedly into smaller subgroups following the same procedure until they represented the most homogeneous subgroups achievable (ie, terminal node). In this study, terminal nodes of these maximum‐sized trees were set to be pure (ie, the node contents only “increase” or “decrease” observations) or with only a single observation. Then a series of subtrees was generated by pruning the initial maximum‐sized trees. To estimate the optimal subtree among the different sized subtrees, 10‐fold cross‐validation analysis was conducted for each subtree followed by the selection based on the one standard error (SE) rule. Briefly, the cross‐validation analysis is used to estimate the risk of misclassification using a randomly selected subset (ie, test samples) of the original dataset (ie, learning sample). The optimal tree is the one that yields the minimal risk estimate. However, the noisy nature of the data and the instability of the cross‐validation procedure can lead to the selection of unstable and large trees.^34^ Therefore, following the one SE rule, the smallest tree that has a cross‐validation risk estimate of less or equal to the minimal risk plus one SE of the minimal error was selected as the optimal tree^30^, ^35^ (Figures S1 and S2).

## 3 RESULTS

**Figure 2:**
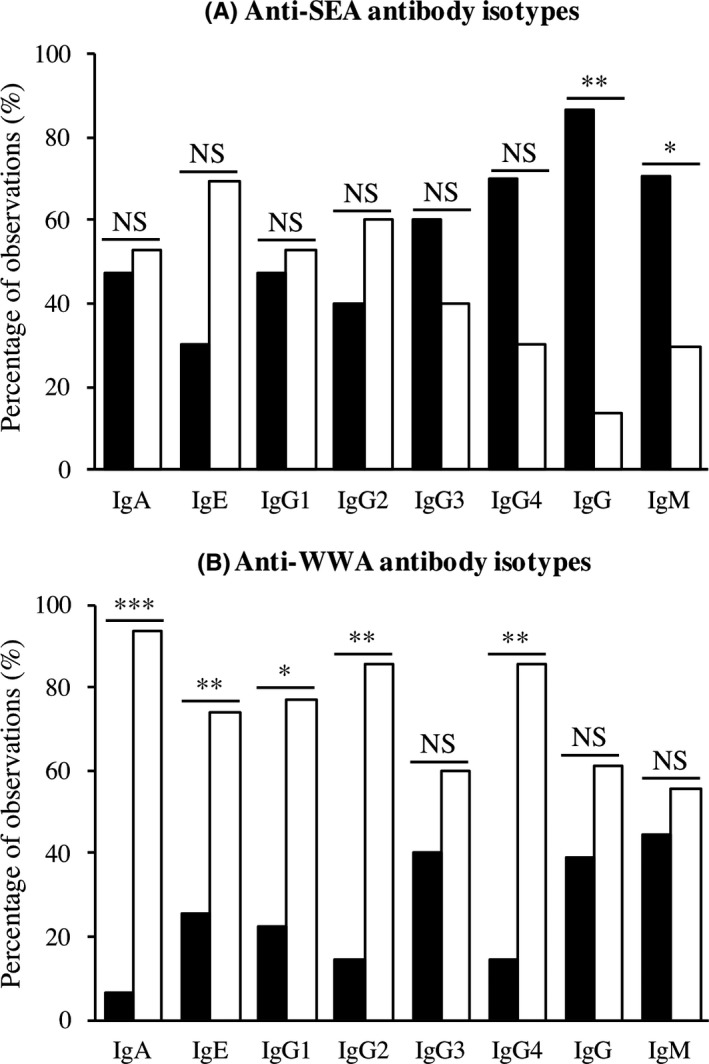
The percentage of observations with increasing or decreasing levels of (A) anti‐SEA, and (B) anti‐WWA antibody isotypes after praziquantel treatment for eight antibody isotypes. The graph shows the fraction of observations that reported a decrease/no change (filled bar) or an increase (unfilled bar) in each antibody isotype. Chi‐square tests were conducted for each pair of anti‐SEA or anti‐WWA antibody isotype. NS non‐significant, *significant at p < 0.05, **significant at *P* < 0.01, ***significant at *P* < 0.001

Following a systematic review, a total of 92 observations from 26 articles (published 1988‐2013) met all inclusion criteria and were considered for the final meta‐analysis. There was a high degree of heterogeneity in the direction of change of antibodies (increase/decrease) after PZQ treatment depending on population studied (Figure 2). The results showed a tendency towards an increase in anti‐WWA antibodies, in contrast with no significant tendency or decrease in anti‐SEA antibodies post‐PZQ treatment (Figure 2). Five anti‐WWA antibody isotypes (IgA, IgE, IgG1, IgG2, IgG4) showed a significant trend of increase (*X* ^2^ = 12.25, *P* < 0.001; *X* ^2^ **= 8.26, *P* = 0.004; *X* ^*2*^ **= 6.55, *P* = 0.011; *X* ^*2*^ = 7.14, *P* = 0.008; *X* ^2^ = 10.71, *P* = 0.001, respectively) (Figure 2). In contrast, two anti‐SEA antibody isotypes (IgG, IgM) showed a significant trend of decrease after PZQ treatment (*X* ^*2*^ **= 8.07, *P* = 0.005; *X* ^*2*^ **= 4.48, *P* = 0.034, respectively) (Figure 2). The CART used to determine predictors of antibody level change identified age/infection intensity as the most common factor affecting post‐treatment change. Thus, the analysis identified optimal trees for anti‐SEA (IgA, IgE, IgG1, IgG2, IgM) and for anti‐WWA (IgG, IgM) (Table 3). On the other hand, none of the predictors included in the analyses had a significant overall effect on anti‐SEA (IgG3, IgG4, IgG) and anti‐WWA (IgA, IgE, IgG1, IgG2, IgG3, IgG4) (Table 3).

**Table 3:** Predictors identified by the classification and regression tree model analyses

Predictors	Anti‐SEA antibodies	Anti‐WWA antibodies
Prevalence (%)	‐	‐
Schistosome species	‐	‐
Days after chemotherapy	IgE	
Age/infection intensity	IgA, IgG1, IgG2, IgM	IgG, IgM
No predictor: mostly decrease	IgG3, IgG4, IgG	‐
No predictor: mostly increase	‐	IgA, IgE, IgG1, IgG2, IgG3, IgG4

**Figure 3:**
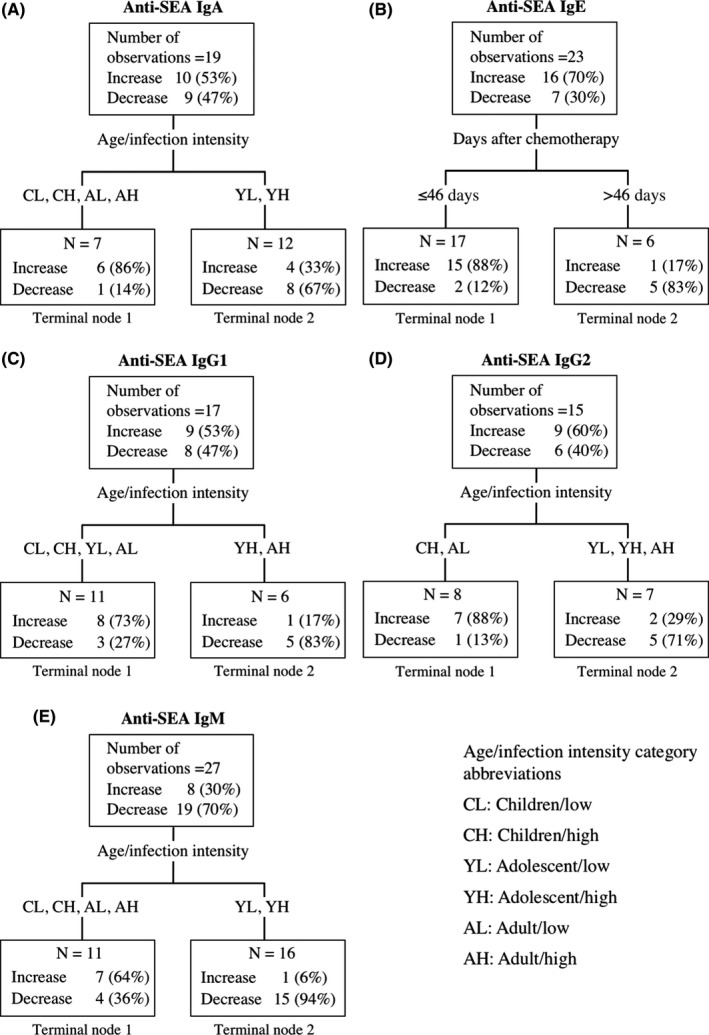
Classification and Regression Tree Models identifying profiles of observations that had higher (increase) or lower (decrease/no change) anti‐SEA antibody isotype levels after praziquantel treatment for (A) anti‐SEA IgA, (B) anti‐SEA IgE, (C) anti‐SEA IgG1, (D) anti‐SEA IgG2 or (E) anti‐SEA IgM. No tree was obtained for the remaining anti‐SEA antibody isotypes. The hierarchy of the Classification and Regression Tree Model starts from the terminal nodes at the top. Abbreviations for age/infection intensity groups are listed in the text box and described in Table2

The first split from the root node of anti‐SEA IgE was based on the number of days post‐PZQ treatment. The proportion of observations that reported increases of anti‐SEA IgE less than 46 days after PZQ treatment was 88%, while only 17% of observations reported increases more than 46 days after PZQ treatment (panel B in Figure 3). This result suggests that there is transient increase of anti‐SEA IgE followed by a decrease to below pre‐treatment levels.

**Figure 4:**
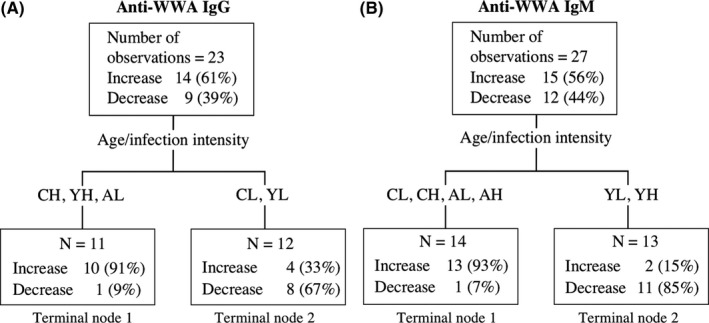
Classification and Regression Tree Models identifying profiles of observations that had higher (increase) or lower (decrease/no change) anti‐WWA antibody isotype levels after praziquantel treatment for (A) anti‐WWA IgG or (B) anti‐WWA IgM. No tree was obtained for the remaining anti‐WWA antibody isotypes. The hierarchy of the Classification and Regression Tree Model starts from the terminal nodes at the top. Abbreviations for age/infection intensity groups are as listed in the text box in Fig 3, also described in Table 2

**Figure 5:**
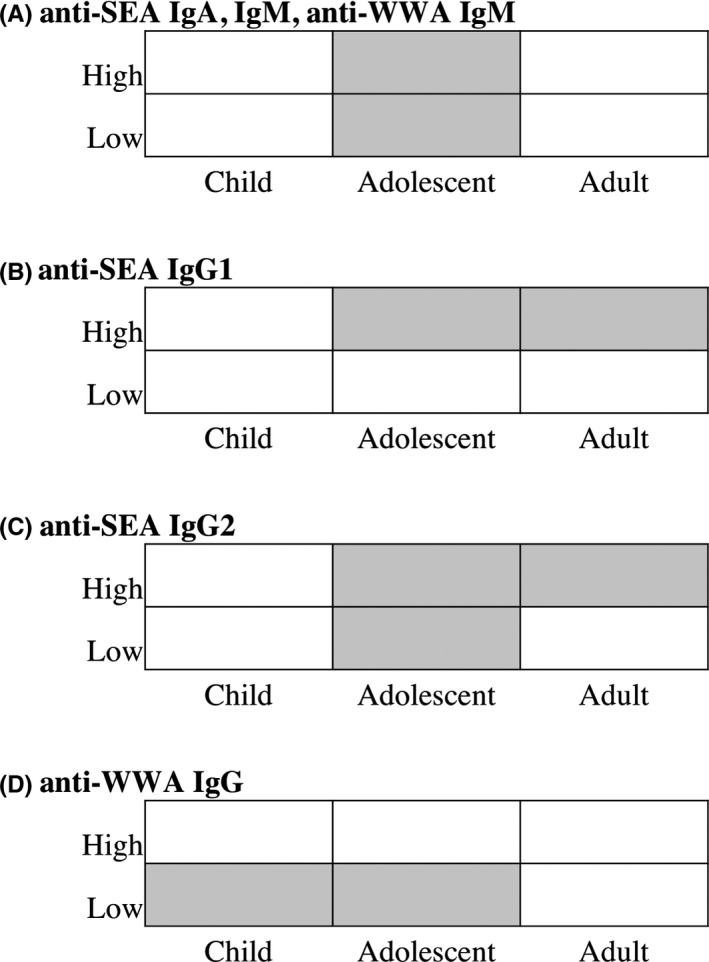
The influence of age and pre‐treatment infection intensity for the direction of change of some antibody isotypes. The graph shows tendency of decrease (filled cell) or increase (unfilled cell) of each antibody isotype by age and pre‐treatment infection intensity for (A) anti‐SEA IgA, IgM, and anti‐WWA IgM, (B) anti‐SEA IgG1, (C) anti‐SEA IgG2 and (D) anti‐WWA IgG

The composite predictor age/infection intensity was identified as the most influential variable for direction of change for anti‐SEA antibodies (IgA, IgG1, IgG2, IgM) and for anti‐WWA (IgG, IgM) antibodies (panels A, C, D, E in Figures 3 and 4). Anti‐SEA IgG1 showed a tendency to a decrease among adolescents and adults with high pre‐treatment infection but an increase among all other age and infection intensity groups (panel B in Figure 5). Anti‐WWA IgG showed the tendency to a decrease among children and adolescents with low pre‐treatment infection intensity but an increase among all other age and infection intensity groups (panel D in Figure 5). The CART results showed the tendency of anti‐SEA (IgA, IgM) and anti‐WWA IgM to increase among children and adults, but decrease among adolescents regardless of their pre‐treatment infection intensity (panel A in Figure 5). Similarly, the results showed a tendency of anti‐SEA IgG2 to decrease among adolescents and increase among children regardless of their pre‐treatment infection intensity. In addition, the results also showed that anti‐SEA IgG2 tends to increase among adults with low pre‐treatment infection intensity but decrease among adults with high pre‐treatment infection intensity (panel C in Figure 2). Overall, changes in levels of anti‐SEA IgG1, IgG2 and anti‐WWA IgG after PZQ treatment were influenced by the combination of age and pre‐treatment infection intensity. On the other hand, anti‐SEA IgA, IgM and anti‐WWA IgM were influenced by age only (Figure 5).

## 4 DISCUSSION

Praziquantel (PZQ) is currently the recommended drug for treatment of schistosomiasis.^1^ Immunology field studies have reported that PZQ treatment can enhance the development of host protective immunity against future re‐infection.^28^, ^36^ Schistosome parasite‐specific antibodies are thought to play an important role in this protective immunity.^37^, ^38^, ^39^ As schistosomiasis endemic areas see increasing PZQ treatment in Mass Drug Administration (MDA) programs, it is important to determine any patterns in population‐wide changes in immune responses and their drivers. Here, we conducted a systematic review and meta‐analysis to identify predictors that influence the direction of change in schistosome‐specific antibody isotype levels after PZQ treatment in humans.

Our analyses showed that the antibodies’ direction of change after chemotherapy is highly variable among different populations. There was only single antibody isotype: anti‐WWA IgA that have been reported to increase after PZQ treatment in all the human populations studied. Our analyses also showed that the antibodies to whole worm antigens (WWA) being reported to increase in the majority of studies. In contrast, studies of anti‐SEA antibody isotypes showed a weak tendency towards decreased levels after PZQ treatment. PZQ treatment has been reported to damage adult worm tegument, therefore allowing the host immune system to detect schistosome worm antigens that would otherwise not be accessible until those worms die naturally.^5^, ^15^ This theory is in line with a previous report that PZQ treatment enhances the host immunological recognition of *S haematobium* specific proteins.^15^ The results of the present study are consistent with this hypothesis and that the elevation of some anti‐WWA antibodies (IgA, IgE, IgG1, IgG2, IgG4) is less likely to be affected by characteristics of the populations.

There are multiple schistosome‐specific antibodies whose association with protection for schistosomiasis has been reported. In particular, schistosome specific IgE and IgA are commonly associated with protection against re‐infection after PZQ treatment in humans.^40^, ^41^, ^42^ Ideally, vaccination programs would be the most effective control strategy for schistosomiasis. Nevertheless, although a number of studies have been conducted to develop an effective vaccine for schistosomiasis, we still do not have any licensed schistosome vaccine. A review of animal studies has shown that attenuated schistosome parasite vaccines are more efficient than recombinant purified protein vaccines.^43^ One potential reason for this improved protection is that attenuated parasite vaccines expose hosts to a large variety of schistosome parasite antigens with a single vaccination and this strategy may provoke an overwhelming response. Similarly, PZQ treatment and damage it does to the worm may release an equivalently large number of antigens that boost acquired protective immunity. Therefore, although the main purpose of PZQ treatment is to cure infected people, it is likely that a beneficial side effect is an increased protection against future re‐infection. In line with this theory, our meta‐analysis shows that indeed, both anti‐WWA IgE and anti‐WWA IgA are elevated after PZQ treatment in the majority (but not all) of study populations. Those reports together with our anti‐WWA IgE and IgA results confirm the “booster” effect of PZQ treatment on a proposed correlate of acquired protective immunity among schistosome affected populations.

There are a number of studies that have reported the potential immunizing effect of PZQ treatment among affected populations. Nevertheless, the length of protection that could be induced by PZQ treatment remains unclear. Our analysis demonstrated the tendency for both anti‐WWA IgE and anti‐WWA IgA levels to be elevated during a 180‐day period after treatment which suggests at least a 6 month protective window. Naus et al. (1998) conducted an immunological study among Cameroonian children and found that anti‐WWA IgE levels were elevated 1 month after the PZQ treatment but then became lower than pre‐treatment baseline level 12 months after the treatment.^44^ That report together with our results indicates that the immunizing effect induced by PZQ treatment can last for as long as 6 months post‐treatment, but may not last longer than 12 months. Further epidemiological studies are therefore required to confirm the dynamics of anti‐WWA IgE and IgA after PZQ treatment to estimate the length of this induced protective immunity. These results also highlight the continuing need for the development of vaccines that can yield long‐lasting protection and preclude infection as a requirement for immunity.

The analysis revealed a significant proportion of studies showing an increase rather than decrease in anti‐WWA IgG4 (86% of observations reported increase) after treatment. Longitudinal and cross‐sectional population studies have demonstrated the association of both anti‐SEA IgG4 and anti‐WWA IgG4 with human susceptibility to re‐infection after treatment in schistosomiasis endemic areas.^45^, ^46^ In particular, IgG4 has been suggested as a possible blocking antibody that inhibits the action of protective IgE in both *S haematobium* and *S mansoni* infections.^39^, ^47^, ^48^ Field studies have reported that the ratio of IgE to IgG4 has a positive influence on resistance to future *S mansoni* re‐infection.^49^, ^50^ There were multiple observations that reported the direction of change for both IgE and IgG4 after praziquantel treatment. The half of those anti‐SEA studies (nine observations) have reported the post‐treatment increase of IgE and decrease of IgG4, supporting the immunizing effect of praziquantel treatment. On the other hand, the majority of anti‐WWA studies reported the post‐treatment increase of both IgE and IgG4. More research is required to determine how the ratio of these two antibodies changes after chemotherapy, and how they relate to protection against re‐infection.

The CART analysis results showed that for several antibody isotypes, the direction of change after chemotherapy can be partially explained by a combination of the population's age and pre‐treatment infection intensity. Pre‐treatment infection intensity determines the quantity of schistosome parasites’ antigens that participants are exposed to following PZQ treatment. On the other hand, in endemic areas, participant age correlates with the cumulative history of exposure to schistosome parasites.^51^ A study in baboons reported that attenuated schistosome parasite vaccines were effective regardless of the different schistosome infection history (naïve or infected then PZQ‐treated).^52^ Supporting this, our results suggest that some isotypes that offer putative protection against re‐infection (anti‐WWA IgA and IgE) are boosted by praziquantel treatment for a minimum of 6 months post treatment regardless of age. However, for other putatively protective isotypes (anti‐SEA IgA) our results do indicate an effect of age. Overall, our result suggests that when schistosome parasite vaccine for human use becomes available, the effectiveness of the vaccine might be affected by not only vaccination dose but also by the age/infection history of participants. Effective vaccine development would benefit from investigating the immunological variability of the target human population.

There are limitations of the current study. First of all, CART analysis does not allow the random effects to be taken into account. Therefore, although multiple observations were extracted from a single article, random effect was not considered in the current study. A preliminary analysis was conducted using mixed effects logistic regression models, which allowed random effect in the model. However, these models were not considered further. This was mainly because the majority of the models had very high information criterion values, indicating instability (results are not shown). Additionally, there are some previously identified candidate predictors such as sex^21^ and co‐infection with human immunodeficiency virus (HIV),^22^ which could not be considered in the current study due to the fact that only a limited number of reports were available.

This meta‐analysis revealed that literature studies, on the whole, tended to report an increase of anti‐WWA antibodies isotypes after PZQ treatment. Our analyses also show a considerable variability in change among different antigens, antibody isotypes and populations following treatment with PZQ, confirming the work of Mutapi (2001).^18^ Although the combination of age and infection intensity, alongside the number of days after treatment, were identified as influential predictors for some antibody isotypes, there is no single predictor that consistently affects all antibody isotypes in the same way. Our results also demonstrated that antibody isotypes that have been reported to have a protective effect against future re‐infection (anti‐WWA IgA, IgE) can be stimulated by PZQ treatment in the majority of cases for a minimum of 6 months after treatment. These results, therefore, reinforce previously reported protection enhancement following PZQ treatment.

## DISCLOSURES

None.

## References

[1] WHO . Schistosomiasis Fact sheet N°115. 2015; http://www.who.int/mediacentre/factsheets/fs115/en/. Accessed Aug., 2015.

[2] Cleland CR , Tukahebwa EM , Fenwick A , Blair L . Mass drug administration with praziquantel reduces the prevalence of *Schistosoma mansoni* and improves liver morbidity in untreated preschool children. Trans R Soc Trop Med Hyg. 2014;108(9):575‐581. 25059523 10.1093/trstmh/tru097

[3] Omedo M , Ogutu M , Awiti A , et al. The effect of a health communication campaign on compliance with mass drug administration for schistosomiasis control in Western Kenya ‐ the score project. Am J Trop Med Hyg. 2014;91(5):982‐988. 25246690 10.4269/ajtmh.14-0136 PMC4228896

[4] Tuhebwe D , Bagonza J , Kiracho EE , Yeka A , Elliott AM , Nuwaha F . Uptake of mass drug administration programme for schistosomiasis control in Koome Islands, Central Uganda. PLoS ONE. 2015;10(4):e0123673. 25830917 10.1371/journal.pone.0123673 PMC4382187

[5] Harnett W , Kusel JR . Increased exposure of parasite antigens at the surface of adult male *Schistosoma mansoni* exposed to praziquantel invitro. Parasitology. 1986;93(2):401‐405. 2431374 10.1017/s0031182000051568

[6] Doenhoff MJ , Cioli D , Utzinger J . Praziquantel: mechanisms of action, resistance and new derivatives for schistosomiasis. Curr Opin Infect Dis. 2008;21(6):659‐667. 18978535 10.1097/QCO.0b013e328318978f

[7] Woolhouse ME , Hagan P . Seeking the ghost of worms past. Nat Med. 1999;5(11):1225‐1227. 10545976 10.1038/15169

[8] Mitchell KM , Mutapi F , Savill NJ , Woolhouse ME . Explaining observed infection and antibody age‐profiles in populations with urogenital schistosomiasis. PLoS Comput Biol. 2011;7(10):e1002237. 22028640 10.1371/journal.pcbi.1002237 PMC3197645

[9] Harris AR , Russell RJ , Charters AD . A review of schistosomiasis in immigrants in Western Australia, demonstrating the unusual longevity of *Schistosoma mansoni* . Trans R Soc Trop Med Hyg. 1984;78(3):385‐388. 6464135 10.1016/0035-9203(84)90129-9

[10] Walter K , Fulford AJC , McBeath R , et al. Increased human IgE induced by killing *Schistosoma mansoni* in vivo is associated with pretreatment Th2 cytokine responsiveness to worm antigens. J Immunol. 2006;177(8):5490‐5498. 17015735 10.4049/jimmunol.177.8.5490

[11] Maizels RM , Yazdanbakhsh M . Immune regulation by helminth parasites: cellular and molecular mechanisms. Nat Rev Immunol. 2003;3(9):733‐744. 12949497 10.1038/nri1183

[12] Mitchell KM , Mutapi F , Savill NJ , Woolhouse MEJ . Protective immunity to *Schistosoma haematobium* infection is primarily an anti‐fecundity response stimulated by the death of adult worms. Proc Natl Acad Sci USA. 2012;109(33):13347‐13352. 22847410 10.1073/pnas.1121051109 PMC3421178

[13] Harnett W . The anthelmintic action of praziquantel. Parasitol Today. 1988;4(5):144‐146. 15463071 10.1016/0169-4758(88)90192-5

[14] Brindley PJ , Sher A . The chemotherapeutic effect of praziquantel against *Schistosoma mansoni* Is dependent on host antibody‐response. J Immunol. 1987;139(1):215‐220. 3108397

[15] Mutapi F , Burchmore R , Mduluza T , et al. Praziquantel treatment of individuals exposed to *Schistosoma haematobium* enhances serological recognition of defined parasite antigens. J Infect Dis. 2005;192(6):1108‐1118. 16107967 10.1086/432553

[16] Schmiedel Y , Mombo‐Ngoma G , Labuda LA , et al. CD4(+) CD25(hi)FOXP3(+) regulatory T cells and cytokine responses in human schistosomiasis before and after treatment with praziquantel. PLoS Negl Trop Dis. 2015;9(8):e0003995. 26291831 10.1371/journal.pntd.0003995 PMC4546370

[17] Van Riet E , Hartgers FC , Yazdanbakhsh M . Chronic helminth infections induce immunomodulation: consequences and mechanisms. Immunobiology. 2007;212(6):475‐490. 17544832 10.1016/j.imbio.2007.03.009

[18] Mutapi F . Heterogeneities in anti‐schistosome humoral responses following chemotherapy. Trends Parasitol. 2001;17(11):518‐524. 11872396 10.1016/s1471-4922(01)02118-3

[19] Rujeni N , Nausch N , Midzi N , Mduluza T , Taylor DW , Mutapi F . Schistosoma haematobium infection levels determine the effect of praziquantel treatment on anti‐schistosome and anti‐mite antibodies. Parasite Immunol. 2012;34(6):330‐340. 22429049 10.1111/j.1365-3024.2012.01363.x PMC3417378

[20] Mutapi F , Hagan P , Woolhouse MEJ , Mduluza T , Ndhlovu PD . Chemotherapy‐induced, age‐related changes in antischistosome antibody responses. Parasite Immunol. 2003;25(2):87‐97. 12791104 10.1046/j.1365-3024.2003.00610.x

[21] Abebe F , Gaarder PI , Petros B , Gundersen SG . Age‐ and sex‐related differences in antibody responses against *Schistosoma mansoni* soluble egg antigen in a cohort of school children in Ethiopia. Apmis. 2001;109(12):816‐824. 11846722 10.1034/j.1600-0463.2001.091203.x

[22] Joseph S , Jones FM , Laidlaw ME , et al. Impairment of the *Schistosoma mansoni*‐specific immune responses elicited by treatment with praziquantel in Ugandans with HIV‐1 coinfection. J Infect Dis. 2004;190(3):613‐618. 15243939 10.1086/422396

[23] Mutapi F , Maizels R , Fenwick A , Woolhouse M . Human schistosomiasis in the post mass drug administration era. Lancet Infect Dis. 2017;17(2):e42‐e48. 27988094 10.1016/S1473-3099(16)30475-3 PMC7614913

[24] Ayeh‐Kumi PF , Addo‐Osafo K , Attah SK , et al. Malaria, helminths and malnutrition: a cross‐sectional survey of school children in the South‐Tongu district of Ghana. BMC Res Notes. 2016;9:242. 27118136 10.1186/s13104-016-2025-3 PMC4847346

[25] Voller A , Bartlett A , Bidwell DE . Enzyme immunoassays with special reference to ELISA techniques. J Clin Pathol. 1978;31(6):507‐520. 78929 10.1136/jcp.31.6.507 PMC1145337

[26] Van Lieshout L , Stelma FF , Guisse F , et al. The contribution of host‐related factors to low cure rates of praziquantel for the treatment of *Schistosoma mansoni* in Senegal. Am J Trop Med Hyg. 1999;61(5):760‐765. 10586908 10.4269/ajtmh.1999.61.760

[27] Hamadto HH , Rashed SM , el Said A , Elhayawan IA . Humoral and cellular immune response in schistosomiasis pre and post praziquantel therapy. J Egypt Soc Parasitol. 1990;20(2):667‐672. 2230324

[28] Mutapi F , Ndhlovu PD , Hagan P , et al. Chemotherapy accelerates the development of acquired immune responses to *Schistosoma haematobium* infection. J Infect Dis. 1998;178(1):289‐293. 9652458 10.1086/517456

[29] Loh WY . Classification and regression trees. Wires Data Min Knowl. 2011;1(1):14‐23.

[30] Breiman L . Classification and regression trees. Belmont, Calif: Wadsworth International; 1984.

[31] Borenstein M . Factors that affect precision In: Borenstein M, Hedges LV, Higgins TPJ, Rothstein RH eds. Introduction to meta‐analysis. Chichester, UK: John Wiley & Sons; 2009:51‐55.

[32] Henrard S , Speybroeck N , Hermans C . Classification and regression tree analysis vs. multivariable linear and logistic regression methods as statistical tools for studying haemophilia. Haemophilia. 2015;21(6):715‐722. 26248714 10.1111/hae.12778

[33] Dusseldorp E , van Genugten L , van Buuren S , Verheijden MW , van Empelen P . Combinations of techniques that effectively change health behavior: evidence from Meta‐CART analysis. Health Psychol. 2014;33(12):1530‐1540. 24274802 10.1037/hea0000018

[34] Lewis RJ . An Introduction to Classification and Regression Tree (CART) Analysis. Annual Meeting of the Society for Academic Emergency Medicine; 2000; San Francisco, California.

[35] Lemon SC , Roy J , Clark MA , Friedmann PD , Rakowski W . Classification and regression tree analysis in public health: methodological review and comparison with logistic regression. Ann Behav Med. 2003;26(3):172‐181. 14644693 10.1207/S15324796ABM2603_02

[36] Satti MZ , Lind P , Vennervald BJ , Sulaiman SM , Daffalla AA , Ghalib HW . Specific immunoglobulin measurements related to exposure and resistance to *Schistosoma mansoni* infection in Sudanese canal cleaners. Clin Exp Immunol. 1996;106(1):45‐54. 8870697 10.1046/j.1365-2249.1996.d01-810.x PMC2200570

[37] Dunne DW , Butterworth AE , Fulford AJ , et al. Immunity after treatment of human schistosomiasis: association between IgE antibodies to adult worm antigens and resistance to reinfection. Eur J Immunol. 1992;22(6):1483‐1494. 1601036 10.1002/eji.1830220622

[38] Zhang Z , Wu H , Chen S , et al. Association between IgE antibody against soluble egg antigen and resistance to reinfection with *Schistosoma japonicum* . Trans R Soc Trop Med Hyg. 1997;91(5):606‐608. 9463682 10.1016/s0035-9203(97)90047-x

[39] Hagan P , Blumenthal UJ , Dunn D , Simpson AJ , Wilkins HA . Human IgE, IgG4 and resistance to reinfection with *Schistosoma haematobium* . Nature. 1991;349(6306):243‐245. 1898985 10.1038/349243a0

[40] Vereecken K , Naus CW , Polman K , et al. Associations between specific antibody responses and resistance to reinfection in a Senegalese population recently exposed to *Schistosoma mansoni* . Trop Med Int Health. 2007;12(3):431‐444. 17313515 10.1111/j.1365-3156.2006.01805.x

[41] Nagaty IM , el Hayawan IA , Nasr ME , el Hamshery AH . Observations on possible immunity to reinfection among school children after schistosomiasis treatment. J Egypt Soc Parasitol. 1996;26(2):443‐452. 8754652

[42] Colley DG , Bustinduy AL , Secor WE , King CH . Human schistosomiasis. Lancet. 2014;383(9936):2253‐2264. 24698483 10.1016/S0140-6736(13)61949-2 PMC4672382

[43] Fukushige M , Mitchell KM , Bourke CD , Woolhouse ME , Mutapi F . A meta‐analysis of experimental studies of attenuated I vaccines in the mouse model. Front Immunol. 2015;6:85. 25774157 10.3389/fimmu.2015.00085 PMC4343029

[44] Naus CWA , van Dam GJ , Kremsner PG , Krijger FW , Deelder AM . Human IgE, IgG subclass, and IgM Responses to worm and egg antigens in *Schistosomiasis haematobium:* a 12‐month study of reinfection in Cameroonian children. Clin Infect Dis. 1998;26(5):1142‐1147. 9597243 10.1086/520310

[45] Grogan JL , Kremsner PG , van Dam GJ , Deelder AM , Yazdanbakhsh M . Anti‐schistosome IgG4 and IgE at 2 years after chemotherapy: infected versus uninfected individuals. J Infect Dis. 1997;176(5):1344‐1350. 9359737 10.1086/514131

[46] Oliveira RR , Figueiredo JP , Cardoso LS , et al. Factors associated with resistance to *Schistosoma mansoni* infection in an endemic area of Bahia, Brazil. Am J Trop Med Hyg. 2012;86(2):296‐305. 22302866 10.4269/ajtmh.2012.11-0204 PMC3269284

[47] Colley DG , Secor WE . Immunology of human schistosomiasis. Parasite Immunol. 2014;36(8):347‐357. 25142505 10.1111/pim.12087 PMC4278558

[48] Demeure CE , Rihet P , Abel L , Ouattara M , Bourgois A , Dessein AJ . Resistance to *Schistosoma mansoni* in humans ‐ influence of the IgE/IgG4 balance and IgG2 in immunity to reinfection after chemotherapy. J Infect Dis. 1993;168(4):1000‐1008. 7690821 10.1093/infdis/168.4.1000

[49] Pinot de Moira A , Fulford AJ , Kabatereine NB , Ouma JH , Booth M , Dunne DW . Analysis of complex patterns of human exposure and immunity to *Schistosomiasis mansoni*: the influence of age, sex, ethnicity and IgE. PLoS Negl Trop Dis 2010;4(9):e820. 20856909 10.1371/journal.pntd.0000820 PMC2939029

[50] Ndombi EM , Abudho B , Kittur N , et al. Effect of four rounds of annual school‐wide mass praziquantel treatment for schistosoma mansoni control on schistosome‐specific immune responses. Parasite Immunol. 2018;40(6):e12530. 29604074 10.1111/pim.12530 PMC6001474

[51] Ruganuza DM , Mazigo HD , Waihenya R , Morona D , Mkoji GM . *Schistosoma mansoni* among pre‐school children in Musozi village, Ukerewe Island, North‐Western‐Tanzania: prevalence and associated risk factors. Parasite Vector. 2015;8:377. 10.1186/s13071-015-0997-9 PMC4504164 26178484

[52] Kariuki TM , Farah IO . Resistance to re‐infection after exposure to normal and attenuated schistosome parasites in the baboon model. Parasite Immunol. 2005;27(7–8):281‐288. 16138849 10.1111/j.1365-3024.2005.00783.x

