# Supplementary figures and images for: Population level changes in schistosome‐specific antibody levels following chemotherapy
Source: Parasite Immunol. 2018 Dec 19;41(1):e12604. doi: 10.1111/pim.12604 (PMC6492179; doi:10.1111/pim.12604)

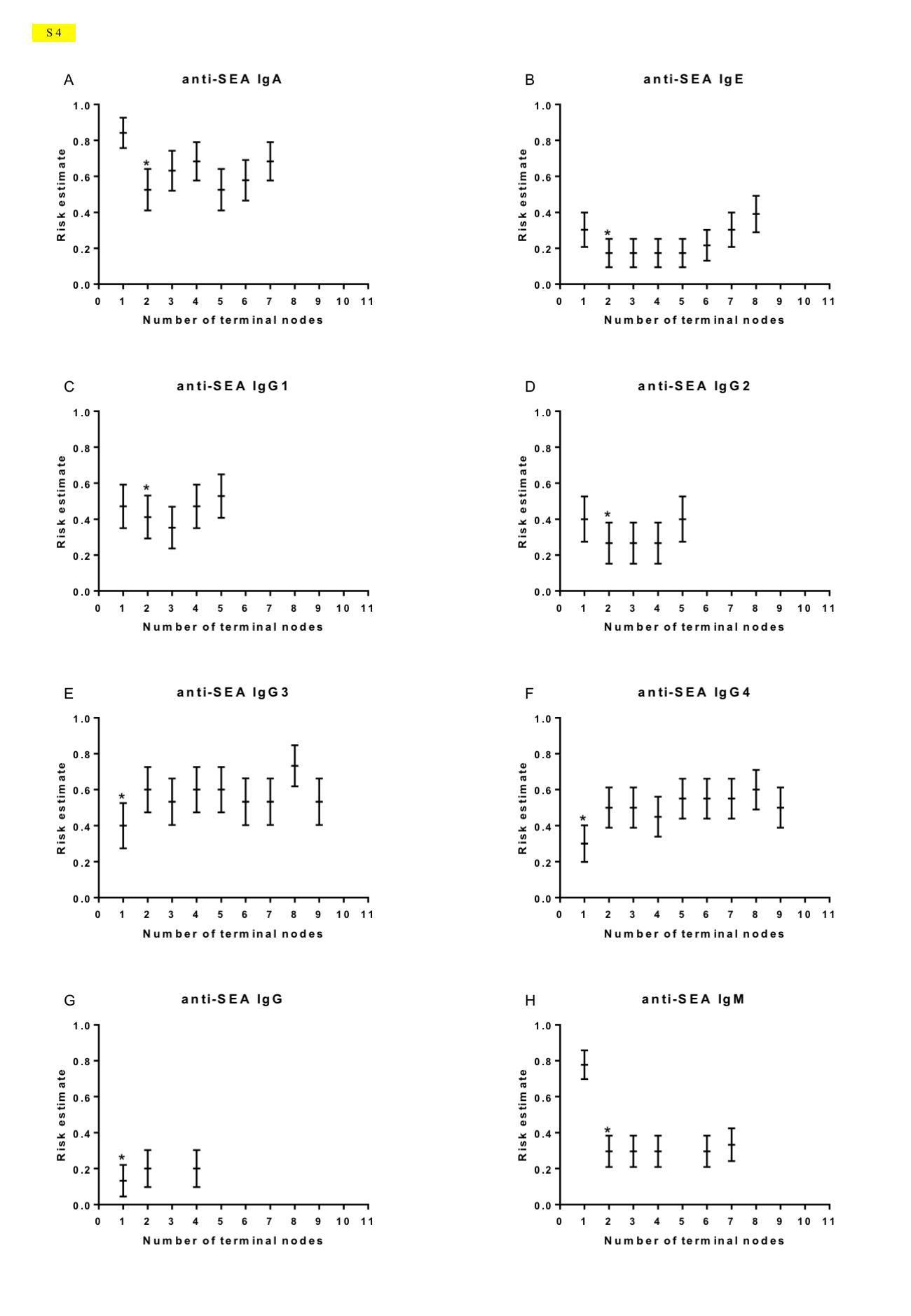

Supplement: Supplementary file 1 [file PIM-41-na-s001.tiff]

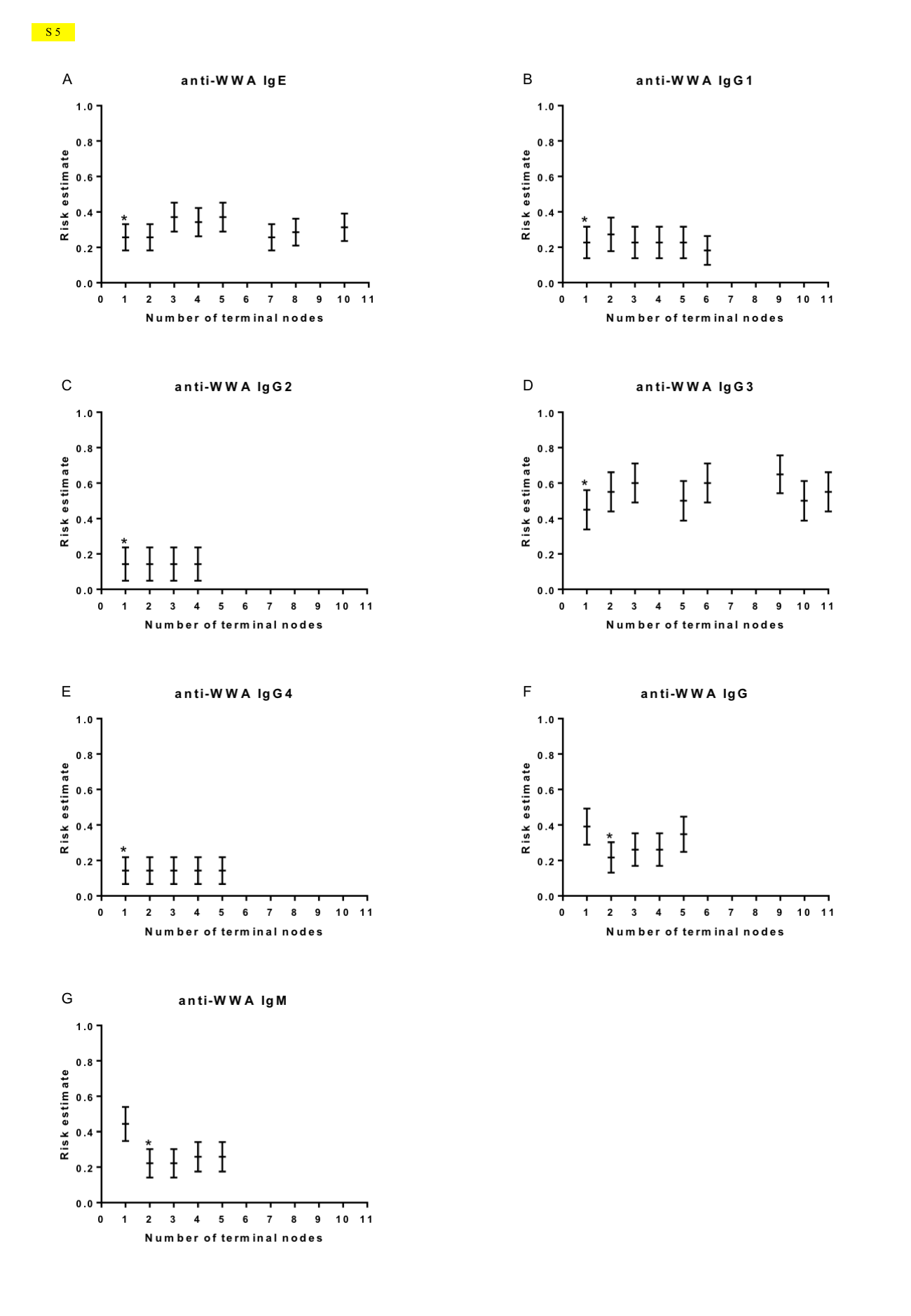

Supplement: Supplementary file 2 [file PIM-41-na-s002.tiff]
