# Supplementary material for: Population level changes in schistosome‐specific antibody levels following chemotherapy
Source: Parasite Immunol. 2018 Dec 19;41(1):e12604. doi: 10.1111/pim.12604 (PMC6492179; doi:10.1111/pim.12604)
Supplement: Supplementary file 3 [file PIM-41-na-s003.docx]

Supporting information

Appendix S1

1. Abebe F, Gaarder PI, et al. Age and sex related differences in antibody responses against *Schistosoma mansoni* soluble egg antigen in a cohort of school children in Ethiopia. *APMIS* (2001) 109(12):816-24. doi: apm091203.

2. Ali AE, Shaheen HI. Human resistance to reinfection with schistosomes. II. Specific IgA titres before & 3 months after praziquantel treatment. *J Egypt Soc Parasitol* (1994) 24(3):505-12.

3. Feldmeier H, Gastl GA, et al. Immune response in chronic *Schistosomiasis haematobium* and *mansoni.* Reversibility of alterations after anti-parasitic treatment with praziquantel. *Scand J Immunol* (1988) 28(2):147-55.

4. Fouda EE, Ali AI, et al. IgE and skin test reactivity in relation to anti-parasitic treatment. *J Allergy Clin Immun* (2007) 119(1):S23-S. doi: DOI 10.1016/j.jaci.2006.11.107.

5. Grogan JL, Kremsner PG, et al. Antischistosome IgG4 and IgE responses are affected differentially by chemotherapy in children versus adults. *J Infect Dis* (1996) 173(5):1242-7.

6. Hamadto HH, Rashed SM, et al. Humoral and cellular immune response in schistosomiasis pre and post praziquantel therapy. *J Egypt Soc Parasitol* (1990) 20(2):667-72.

7. Hussein HM, Kaddah MA, et al. Host and parasite determinants of morbidity in Egyptian children with schistosomiasis. *J Egypt Soc Parasitol* (1996) 26(3):755-72.

8. Ismail MM, Bruce JI, et al. Different immunoglobulin classes and eosinophils as a monitor of cure in schistosomal cases using praziquantel. *J Trop Med* (1992) 2(2):99-108.

9. Joseph S, Jones FM, et al. Impairment of the *Schistosoma mansoni*-specific immune responses elicited by treatment with praziquantel in Ugandans with HIV-1 coinfection. *J Infect Dis* (2004) 190(3):613-8. doi: 10.1086/422396.

10. Mutapi F, Ndhlovu PD, et al. Chemotherapy accelerates the development of acquired immune responses to Schistosoma haematobium infection. *J Infect Dis* (1998) 178(1):289-93.

11. Mutapi F, Ndhlovu PD, et al. Changes in specific anti-egg antibody levels following treatment with praziquantel for *Schistosoma haematobium* infection in children. *Parasite Immunol* (1998) 20(12):595-600.

12. Nagaty IM, el Hayawan IA, et al. Observations on possible immunity to reinfection among school children after schistosomiasis treatment. *J Egypt Soc Parasitol* (1996) 26(2):443-52.

13. Nassr A, Hassan MM, et al. IgG isotypes in schistosomiasis patients before and after praziquantel. *J Egypt Soc Parasitol* (2002) 32(3):931-52.

14. Naus CWA, van Dam GJ, et al. Human IgE, IgG subclass, and IgM Responses to worm and egg antigens in *Schistosomiasis haematobium*: A 12-month study of reinfection in Cameroonian children. *Clin Infect Dis* (1998) 26(5):1142-7.

15. Reilly L, Magkrioti C, et al. Effect of treating *Schistosoma haematobium* infection on *Plasmodium falciparum*-specific antibody responses. *BMC Infect Dis* (2008) 8(158). doi: Doi 10.1186/1471-2334-8-158.

16. Satti MZ, Cahen P, et al. Changes in IgE- and antigen-dependent histamine-release in peripheral blood of *Schistosoma mansoni*-infected Ugandan fishermen after treatment with praziquantel. *BMC immunology* (2004) 5(6). doi: 10.1186/1471-2172-5-6.

17. Satti MZ, Lind P, et al. Specific immunoglobulin measurements related to exposure and resistance to *Schistosoma mansoni* infection in Sudanese canal cleaners. *Clin Exp Immunol* (1996) 106(1):45-54. doi: DOI 10.1046/j.1365-2249.1996.d01-810.x.

18. Satti MZ, Sulaiman SM, et al. Clinical, parasitological and immunological features of canal cleaners hyper-exposed-to *Schistosoma mansoni* in the Sudan. *Clin Exp Immunol* (1996) 104(3):426-31. doi: DOI 10.1046/j.1365-2249.1996.00051.x.

19. Snyman JR, de Sommers K, et al. Effects of calcitriol on eosinophil activity and antibody responses in patients with schistosomiasis. *Eur J Clin Pharmacol* (1997) 52(4):277-80.

20. Snyman JR, Sommers de K. Effect of levamisole on the immune response of patients with schistosomiasis after treatment with praziquantel. *Clin Drug Investig* (1998) 15(6):483-9. 21. Tweyongyere R, Mawa PA, et al. Effect of praziquantel treatment of *Schistosoma mansoni* during pregnancy on intensity of infection and antibody responses to schistosome antigens: results of a randomised, placebo-controlled trial. *Bmc Infect Dis* (2009) 9(32). doi: 10.1186/1471-2334-9-32.

22. van Lieshout L, Stelma FF, et al. The contribution of host-related factors to low cure rates of praziquantel for the treatment of *Schistosoma mansoni* in Senegal. Am J Trop Med Hyg (1999) 61(5):760-5.

23. Vereecken K, Naus CWA, et al. Associations between specific antibody responses and resistance to reinfection in a Senegalese population recently exposed to *Schistosoma mansoni*. *Trop Med Int Health* (2007) 12(3):431-44.

24. Walter K, Fulford AJC, et al. Increased human IgE induced by killing *Schistosoma mansoni* in vivo is associated with pretreatment Th2 cytokine responsiveness to worm antigens. *J Immunol* (2006) 177(8):5490-8.

25. Wilson S, Jones FM, et al. Rapidly boosted plasma Il-5 induced by treatment of human *Schistosomiasis haematobium* is dependent on antigen dose, ige and eosinophils. *Plos Neglect Trop D* (2013) 7(3):e2149. doi: 10.1371/Journal.Pntd.0002149.

26. Zinyowera S, Muchaneta-Kubara CE, et al. Changes in the humoral immune responses after chemotherapy in single and co-infected individuals with *Schisosoma haematobium* and *Plasmodium falciparum*. Cent Afr J Med (2006) 52(9-12):104-11.

Table S1

| anti-SEA | N* | days after chemotherapy | anti-WWA | N* | days after chemotherapy |
| --- | --- | --- | --- | --- | --- |
| IgA | 19 | 35-180 | IgA | 16 | 35-180 |
| IgE | 23 | 21-180 | IgE | 35 | 21-180 |
| IgG1 | 17 | 30-126 | IgG1 | 22 | 30-90 |
| IgG2 | 15 | 30-126 | IgG2 | 14 | 30-42 |
| IgG3 | 15 | 30-126 | IgG3 | 20 | 30-63 |
| IgG4 | 20 | 21-126 | IgG4 | 21 | 21-90 |
| IgG | 15 | 35-180 | IgG | 23 | 21-180 |
| IgM | 27 | 30-180 | IgM | 27 | 30-180 |

Table S2

1) Studies that failed to report pre-treatment infection intensity

| Reference | Reference used to obtain infection intensity |
| --- | --- |
| Ali *et al.* 1994 | El-Khoby, T., *et al.* 2000."The epidemiology of schistosomiasis in Egypt: summary findings in nine governorates." Am J Trop Med Hyg 62(2 Suppl): 88-99. |
| Fouda *et al.* 2007 |  |
| Ismail *et al.* 1992 |  |
| Nagaty *et al.* 1996 |  |

| Reference | Reference used to obtain prevalence |
| --- | --- |
| Nassr *et al.* 2002 | Barakat, R. M. 2013."Epidemiology of Schistosomiasis in Egypt: Travel through Time: Review." J Adv Res 4(5): 425-432. |
| Zinyowera *et al.* 2006 | Chimbari, M. J. 2012."Enhancing schistosomiasis control strategy for zimbabwe: building on past experiences." J Parasitol Res 2012: 353768. |

2) Studies that failed to report infection prevalence of the study area
